# Inhibitory Effect of Endostar on Specific Angiogenesis Induced by Human Hepatocellular Carcinoma

**DOI:** 10.1155/2015/957574

**Published:** 2015-04-23

**Authors:** Qing Ye, Shukui Qin, Yanhong Liu, Jundong Feng, Qiong Wu, Wenshu Qu, Xiaojin Yin

**Affiliations:** ^1^Postdoctoral Station of Nanjing General Hospital, Nanjing 210002, China; ^2^PLA Cancer Center in Eighty-One Hospital of People's Liberation Army, Nanjing 210002, China; ^3^Department of Pathology, Affiliated Drum Tower Hospital of Nanjing University Medical School, Nanjing 210008, China; ^4^Jiangsu Harbinger Drug Development Co., Ltd., Nanjing 210002, China

## Abstract

To investigate the effect of endostar on specific angiogenesis induced by human hepatocellular carcinoma, this research systematically elucidated the inhibitory effect on HepG2-induced angiogenesis by endostar from 50 ng/mL to 50000 ng/mL. We employed fluorescence quantitative Boyden chamber analysis, wound-healing assay, flow cytometry examination using a coculture system, quantitative analysis of tube formation, and *in vivo* Matrigel plug assay induced by HCC conditioned media (HCM) and HepG2 compared with normal hepatocyte conditioned media (NCM) and L02. Then, we found that endostar as a tumor angiogenesis inhibitor could potently inhibit human umbilical vein endothelial cell (HUVEC) migration in response to HCM after four- to six-hour action, inhibit HCM-induced HUVEC migration to the lesion part in a dose-dependent manner between 50 ng/mL and 5000 ng/mL at 24 hours, and reduce HUVEC proliferation in a dose-dependent fashion. Endostar inhibited HepG2-induced tube formation of HUVECs which peaked at 50 ng/mL. *In vivo* Matrigel plug formation was also significantly reduced by endostar in HepG2 inducing system rather than in L02 inducing system. It could be concluded that, at cell level, endostar inhibited the angiogenesis-related biological behaviors of HUVEC in response to HCC, including migration, adhesion proliferation, and tube formation. At animal level, endostar inhibited the angiogenesis in response to HCC in Matrigel matrix.

## 1. Introduction

Hepatocellular carcinoma (HCC) is the fifth most common tumor in the world, which is difficult to be cured and easily relapses. Surgical resection, liver transplantation, local interventional therapy, and general chemotherapy were conventional treatments with limitations [1, 2]. It was imperative to find a new systematic therapy for HCC. General chemotherapeutics are aimed at tumor cells, while antiangiogenesis therapeutics are aimed at vascular endothelial cells which have become the second important target because of their stable performance, specificity, and wide scope [3, 4]. HCC is a hyper vascularized tumor, where during it growth cancer cells induce angiogenesis through various pathways.

Abundant blood vessel was observed in HCC angiography, and angiogenesis was closely connected to HCC prognosis [5]. Immunological histological chemistry (IHC) using specific labeled CD34 in vascular endothelial cell to detect microvessel density of HCC was an extensively applied pathological indicator for HCC prognosis [6]. Successful antiangiogenesis therapy in colorectal cancer [7] and lung cancer [8, 9] provides new orientation for HCC antiangiogenesis therapy.

Endothelial cells play important roles in angiogenesis. Among multiple antiangiogenesis factors, endostatin was satisfactory because it is endogenous and multitargeted. Endostatin is the C-terminal amino fragment of type X VIII collagen, which was firstly separated from angioblastoma strains of mouse. Endostatin specifically inhibits proliferation of vascular endothelial cells and capillary growth [10]. It was reported that endostatin could induce cell apoptosis and cell cycle arrest of endothelial cells [11].

Animal experiments showed that endostatin could significantly repress the proliferation of various murine and allogeneic transplantation tumors [12]. However, there were some difficulties in the application of genetic engineering restructuring human endostatin in clinic because restructured endostatin expressed in* E. coli* was in form of inclusion body which was difficult to be purified and refolded [13]. Compared to endostatin, endostar was a newly restructured human vascular endothelial inhibitor with a His tag (MGGSHHHHH) added to its N-terminal which makes it to be purified easily [14]. However, whether this modification influences the biological activity of Endostatin needs further verification.

Some reports and statistics showed that endostar has potential effect on HCC therapy: (a) the density of microvessel in HCC lumps was a prognostic indicator of HCC relapse after surgery [15] and (b) the low expression level of endostatin in HCC and abundant angiogenesis were related to tumor progression [16]. (c) Endostar combined with navelbine and cisplatin in the therapy of another rich vascular tumor, NSCLC, showed that they could improve advanced stage therapy effect and prolong median time of progress of tumors in phases I, II, and III trial [17]. (d) Endostatin containing plasmid showed a suppression inhibitory effect towards human HCC cell line Bel-7402 and its heterogeneous transplantation tumor in nude mice model. (e) Novel angiogenesis inhibitor Avastin combining chemotherapy was in phase II clinic experiments which could prolong the progression free survival (PFS) of HCC patient for 6 months [18].

In this research, we employed HUVEC as the cell model and normal hepatocyte line L02. L02 was used as control with an antiangiogenesis evaluating* in vitro* and* in vivo* experiment system to study the effects of recombinant human endostatin (endostar) on human HCC cell line HepG2-induced angiogenesis-related biological behaviors of HUVECs, which could provide theoretical and experimental evidence for endostar being used as an antiangiogenesis drug in HCC treatment.

## 2. Materials and Methods

### 2.1. Cell Culture

Experiments were performed using HUVECs (ScienCell), HepG2 (from Shanghai Cell Institution), and L02 (from Shanghai Cell Institution). HUVECs were cultured in endothelial cell medium (ECM; ScienCell) supplemented with 1% endothelial cell growth supplement (ECGS; ScienCell), 5% fetal bovine serum (FBS, Invitrogen Corp.), 100 U/mL penicillin (Invitrogen Corp.), and 100 *μ*g/mL streptomycin (Invitrogen Corp.). HepG2 and L02 were cultured in Dulbecco's modified eagle medium (DMEM, Invitrogen Corp.) supplemented with 10% FBS, 100 U/mL penicillin, and 100 *μ*g/mL streptomycin. All the cells were maintained within the 5% CO_2_ air atmosphere in a humidified incubator at 37°C. 90% confluent cells were detached by trypsin/EDTA (Invitrogen Corp.) and were subcultured at proper split ratio.

### 2.2. Animals

BALB c/J-severe combined immunodeficient (SCID) mice were obtained from Shanghai SLAC Laboratory and were used for matrigel plug study.

### 2.3. Preparation of HCM from HepG2 and NCM from L02

As the method described by Moroz et al. [19], cells were allowed to grow till 80% confluence. Following the replacement of the culture medium with 15 mL DMEM plus 0.2% bovine serum albumin (BSA, Invitrogen Corp.), cells were placed within the 5% CO_2_ air atmosphere in a humidified incubator at 37°C for 24 h. HCM or NCM were collected and centrifuged for 15 min at 7,000 rpm to eliminate cell debris. The supernatants were ultra-filtered by Amicon Ultra-15 (Millipore, MWCO 3KD) to obtain protein and were then resuspended in human endothelial serum-free medium (SFM; Invitrogen Corp.) plus 0.2% BSA. The suspension was collected and filtered. The collected medium was stored at −80°C.

### 2.4. Migration Assay

The HUVEC migration assays were performed as described in the protocol using 24-well modified Boyden chambers containing polyethylene membranes (Greiner Bio-One). HUVECs were starved overnight in SFM with 0.2% BSA. The cells were trypsinized, resuspended in SFM containing 0.2% BSA, and mixed with endostar (Simcere Pharmaceutical Co., Ltd.) of different final concentrations (0 ng/mL, 5 ng/mL, 50 ng/m, 500 ng/mL, 5,000 ng/mL, and 50,000 ng/mL) for 30 min before adding the mixture to the upper chamber. HCM and NCM mixed with endostar served as attractants that were placed in the lower wells. SFM with 10% FBS and SFM with 0.2% BSA were employed as positive and negative controls, respectively. The cell culture plates were incubated in a cell culture incubator at 37°C and 5% CO_2_ for 6 h. Subsequently, all cells were fluorescently labeled with 4 *μ*M calcein-AM (Sigma) for 45 min and incubated in prewarmed trypsin-EDTA for 10 min, allowing migratory cells to detach from the underside of the PET membrane. Finally, the migratory cells were quantified in the TECAN multifunctional reader at an excitation wavelength of 485 nm and an emission wavelength of 520 nm.

### 2.5. Wound-Healing Assay

Wound-healing assay was performed as previously described [20] with some improvements. HUVECs (1.8 × 10^4^ per well) were seeded in 24-well plates and cultured in ECM medium to 90% confluence. HUVECs were starved for 4 h in SFM with 0.2% BSA. The cell monolayer was scraped with a sterile tip to create a cell-free zone and then photographed to determine the injury baseline. Media were replaced by HCM containing endostar of different concentration. SFM with 5% FBS and SFM with 0.2% BSA were used as controls. The migration of the cells was recorded at 24 hours on an Olympus IX-71 inverted microscope equipped with an Olympus camera. Data were analyzed by Image-Pro Plus software.

### 2.6. Cell Adhesion Assay

Adhesion between tumor cells and endothelial cells was measured as described with some modifications [21]. HepG2 and L02 were seeded in 96-well plate to 100% confluence before experiments. HUVECs were stained with 4 *μ*M calcein-AM and trypsinized. Single HUVEC suspension was pretreated with endostar of different concentration for 30 min. HUVECs were mixed with HCM before being seeded in the HepG2 cell-coating plate (4 × 10^4^ per well). Plates were incubated at 37°C for 1 h. To remove nonadherent cells, each well was carefully washed by addition of prewarmed serum-free culture medium, followed by gentle swirling and inversion of the plate and blotting of excess liquid onto filter paper or paper towels. This was repeated four times, after which 1 mL of prewarmed serum-free culture medium was added to each well. Relative fluorescence units (RFU) were measured using TECAN multifunctional reader at an excitation wavelength of 485 nm and an emission wavelength of 520 nm. Adhesion rates were calculated by the formula: Adhesion rate = (RFU100%control − RFU0%control)/100 × (RFUsample − RFU0%control).

### 2.7. CFSE-Labeled Flow Cytometric Analysis

The coculture system was established as previously described [22] with some changes. Immediately before the assay, HUVECs and HepG2 were stained with carboxyfluorescein succinimidyl amino ester (CFSE) (Molecular Probes) and PKH-26 red fluorescent cell linker (Sigma-Aldrich), respectively, as the protocol. Parts of the labeled cells were fixed immediately and used as positive control. HUVECs were plated in 12-well plates (4.2 × 10^4^ per well) alone or at ratio of 1 : 4 compared to HepG2. After complete attachment, cells were starved in SFM plus 0.2% BSA for 6 h and treated with endostar for an additional 1 h. Media were replaced by mixture of SFM plus 2% FBS together with endostar of corresponding concentrations. 48 h later, HUVECs were harvested, fixed in cell fixation liquid, and analyzed by flow cytometry. Data were analyzed by CELLQuest and ModFit to obtain proliferation index. Positive control was employed as the first generation. Inhibition rates were calculated by the following formula: IR = (PI_nc_ − PI_sample_)/PI_NC_ × 100%.

### 2.8. Tube Formation Assay

Tube formation was evaluated as previously described [20]. 50 *μ*L of diluted growth factor-reduced Matrigel (Becton Dickinson) was tiled on the bottom of 96-well plates at 4°C and left at 37°C for gelification. HUVECs were seeded at a density of 2.25 × 10^4^ per well in SFM, HCM, and NCM. For coculture experiments, mixtures of HUVECs and HepG2 at a 1 : 1 ratio were seeded on Matrigel in HCM. Endostar of different concentrations was added. All tube formation experiments were observed using Olympus IX-71 inverted fluorescence microscopy and images were digitally captured at 24 hours after plating. Tubule formation was assessed by counting the number of tubule branches and the total area covered by tubules in each field of view by Image-Pro Plus software.

### 2.9. *In Vivo* Matrigel Plug Assay

Angiogenesis was analyzed using the* in vivo* Matrigel plug assay as previously described [23]. Briefly, GFR-Matrigel, HepG2/L02, and endostar at a 2 : 1 : 1 volume ratio were thoroughly mixed at 4°C. Male BALB/c SCID mice were anesthetized and injected with the mixture of GFR-Matrigel and HepG2 subcutaneously (s.c.) on the upper left dorsal. GFR-Matrigel and L02 cell mixture was injected on the upper right part using a prechilled tuberculin syringe (27-gauge needle). The mixture of GFR-Matrigel and endostar was employed as blank control and was injected on the bottom left dorsal. Starting from the second day, 8 mg/kg endostar was given to mice every day by intraperitoneal injection. Seven days after implantation, mice were sacrificed. Matrigel plugs with the surrounding skin were removed and vascularity was photographed (37). Then Matrigel plugs were homogenized in 500 *μ*L of radioimmunoprecipitation (RIPA; Beyotime) buffer. The homogenate was centrifuged at 1,000 ×g to collect the suspension. OD values of Hemoglobin were measured at the absorbance wavelength 420 nm using a TECAN multifunctional reader. Results were analyzed by ANVOA test.

## 3. Result

### 3.1. The Effects of Endostar on HCC-Induced HUVEC Migration

**Figure 1:**
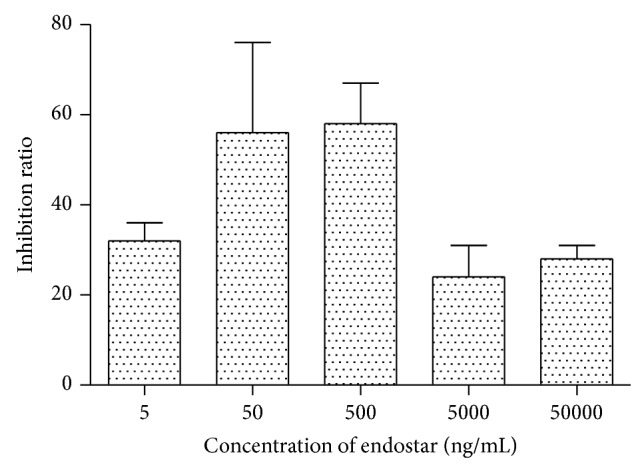
Effect of endostar towards HUVEC chemotaxis induced by HCM.

Boyden chambers migration assay showed that when using HCM as attractants, migration of HUVEC was obviously induced in 4–6 h which could be inhibited by endostar with concentration of 5 ng/mL to 50,000 ng/mL. When the concentration of endostar was 50 ng/mL and 500 ng/mL, the suppression effect was most significant (*P* < 0.01) (Figure 1).

**Figure 2:**
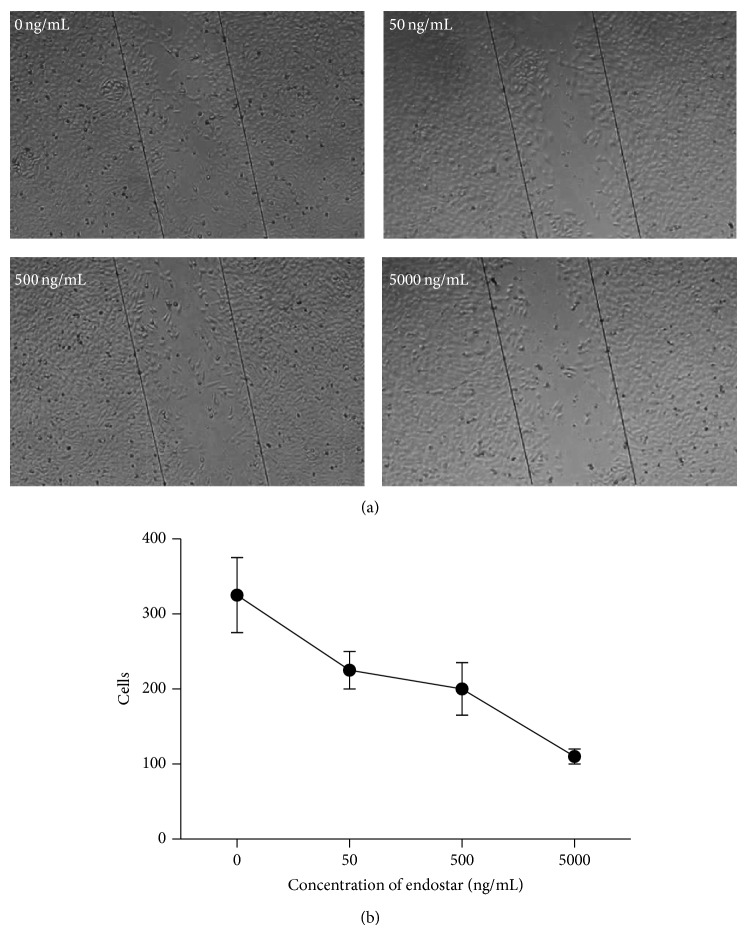
HCM induced migration of HUVEC to wound area was suppressed by endostar. (a) Endostar with different concentrations suppressed HUVEC migration detected by microscopy. (b) The amount of HUVEC cells migration suppressed by different concentrations of endostar.

Wound-healing assay showed that HCM induced migration of HUVEC to wound area which could be suppressed by endostar (Figure 2(a)). After 24 h, the suppression effect of endostar was concentration-dependent from the concentration of 50 ng/mL to 5,000 ng/mL (Figure 2(b)).

### 3.2. The Effects of Endostar on HCC-Induced HUVEC Adhesion

**Figure 3:**
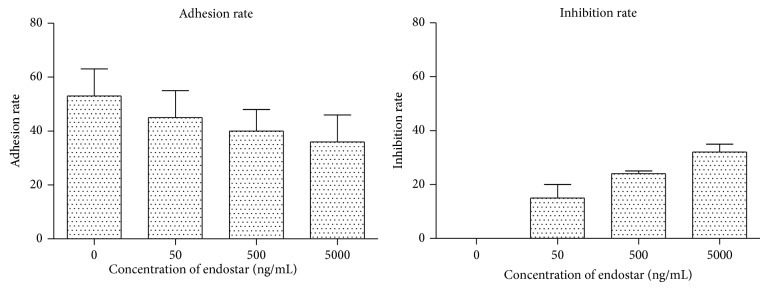
Adhesion rate and suppression rate effect by different endostar concentrations in cell adhesion assay.

In cell adhesion assay, adhesion rate and suppression rate of each group were calculated by RFU as shown in Figure 3. It was revealed that the cell number of HUVEC that adhered to HepG2 was decreased by addition of endostar. The suppression effect of endostar towards adhesion ability of HUVEC to HepG2 increased as its concentration elevated from 50 ng/mL to 5,000 ng/mL.

### 3.3. The Effects of Endostar on HCC-Induced HUVEC Proliferation

**Figure 4:**
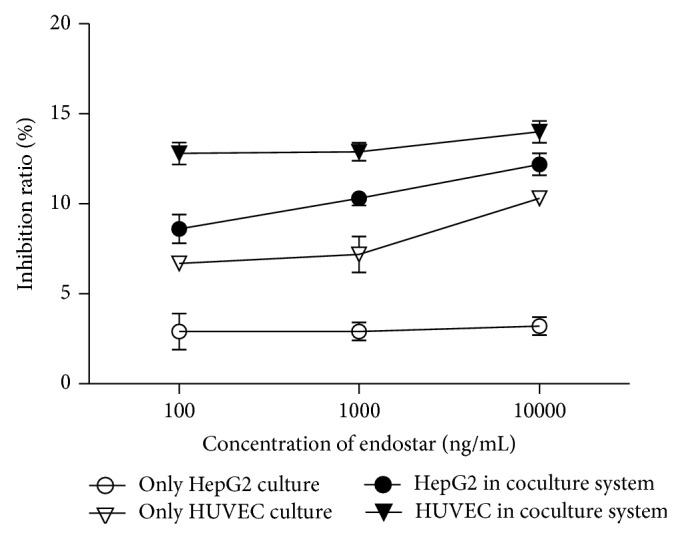
The effects of different concentrations of endostar on HCC-induced HUVEC proliferation detected by CFSE-labeled flow cytometric analysis.

The result of CFSE-labeled flow cytometric analysis was analyzed by CELLQuest and ModFit and the PI value of each sample was calculated and exhibited in Figure 4, which suggested that endostar significantly suppressed the proliferation of HUVEC in HepG2 cocultured system and HCM culture system dose-dependently. Furthermore, the suppression effect of endostar towards the proliferation of HUVEC was more significant in coculture system than in HCM system (*P* < 0.05).

### 3.4. The Effects of Endostar on HCC-Induced Tube Formation

**Figure 5:**
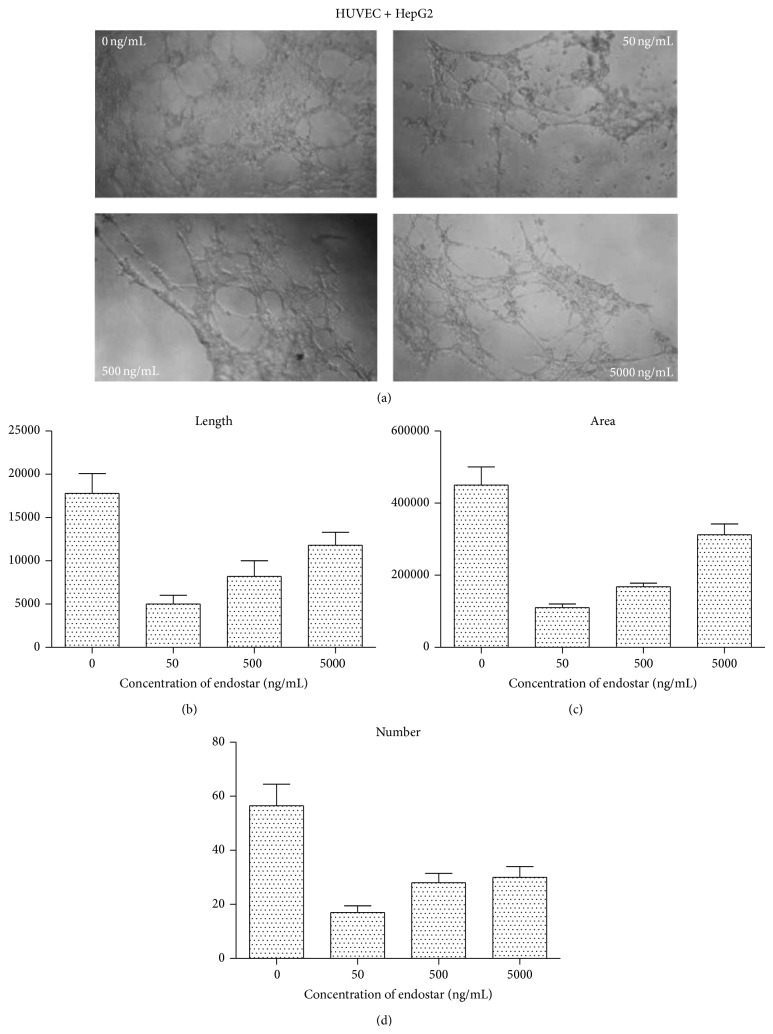
Tube formation of HUVEC induced by HCM was suppressed by endostar. (a) Endostar suppressed tube formation of HUVEC induced by HCM detected by microscopy. (b) Endostar suppressed the length of formatted net tube. (c) Endostar suppressed the area of formatted net tube. (d) Endostar suppressed the amount of formatted net tube.

Tube formation assay showed that the HUVEC net tube structure induced by HepG2 was significantly decreased by addition of endostar with different concentrations (Figure 5(a)). Analysis with Image-Pro Plus showed that the length (Figure 5(b)), area (Figure 5(c)), and amount (Figure 5(d)) of Matrigel net tube formed by HUVEC all decreased after endostar administration. The suppression effect was most significant when the concentration was 50 ng/mL.

### 3.5. The Effects of Endostar on HCC-Induced Angiogenesis* In Vivo*

**Figure 6:**
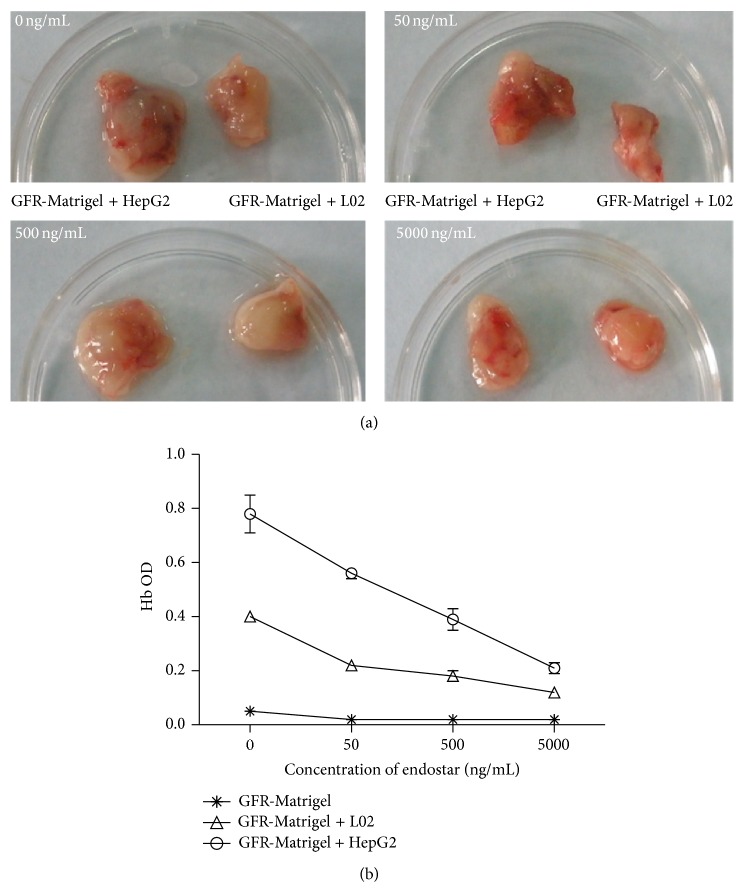
The suppression effect of endostar towards HCC-induced angiogenesis detected by* in vivo* Matrigel plug assay. (a) The Matrigel plugs formatted in SCID mouse. (b) The quantity of homogenate in Matrigel plugs.

*In vivo* Matrigel plug assay showed that GFR-Matrigel + HepG2, GFR-Matrigel + L02 could form plug containing initial vessel in subcutaneous SCID mouse. The number of initial vessels in the plug was decreased by endostar. In Figure 6(a), when the concentration of endostar was 0 ng/mL, there were a large number of initial vessels in the plug formed by GFR-Matrigel + HepG2 and the color of the plug was dark red. When the concentration of endostar was 50 ng/mL, the initial vessels were significantly decreased except a huge initial vessel in the plug. When the concentration of endostar was 500 ng/mL, there were some tiny vessels left in the plug. When the concentration of endostar was 5,000 ng/mL, the color of plug was pale. After homogenization, the homogenate OD values of endostar-treated plugs decreased which suggested that endostar significantly suppressed HepG2-induced angiogenesis (Figure 6(b)).

## 4. Discussion

The mechanism of HCC-induced endothelial cell migration was generally considered to be related to various cytokines of HCC cells such as vascular endothelial growth factor (VEGF) family, fibroblast growth factors (FGF) family, Angiopoietin2, Angiotropin, tumor necrosis factor (TNF)-*α*, colony stimulating factors (CSFs), CXC chemokines with ELR motif, hepatocyte growth factor (HGF), platelet endothelial cell adhesion molecule- (PECAM-) 1, and Integrins [24]. A network was established by interaction of these cytokines; however, the weight of each cytokines was unknown. In general, endostar could inhibit chemotactic migration of endothelial cells induced by the multiple cytokines secreted by HCC cells. In fluorescence quantitative migration chamber experiments, multiple cytokines induced chemotactic migration of endothelial cells, which was inhibited after endostar was added. When endostar was added for 4 h to 6 h with concentrations from 5 ng/mL to 50,000 ng/mL, inhibitory effect was expressed and it was most significant with concentrations of 50 ng/mL and 500 ng/mL.

Similar results were detected in scratch experiment. After scratch, HCM induced chemotactic migration of endothelial cells to injured part. When endostar was added for 24 h, with the concentrations from 5 ng/mL to 50,000 ng/mL, the inhibition of chemotactic migration ability of endothelial cells to injured part by HCM was concentration-dependent. Although the two experiments suggested that endostar could inhibit endothelial cell migration induced by HCM, the most effective concentration was different, which was dependent on different action time of endostar.

The adhesion mechanism of endothelial cell induced by HCC cells was generally considered to be related to the secretion of VEGF, FGF-2 by HCC cells, and integrins (*α*1*β*1 and *α*2*β*1), VE-cadherin, and PECAM-1 upregulated in endothelial cells, which could improve the adhesion effect of endothelial cells to HCC cells [25]. Endostar can effectively suppress the adhesion of endothelial cell induced by HCC, which was detected by the experiment of fluorescent quantitative adhesion. The number of HUVEC cells that were activated by HCM and that adhered to HepG2 significantly decreased when endostar was added. When the concentration of endostar was from 5 ng/mL to 50,000 ng/mL, the adhesion suppression effect was concentration-dependent.

HCC cells induce the proliferation of vessel endothelial cell via two approaches: one was secreting some cytokines such as VEGF-family, FGF-family, angiopoietin-2, EGF, CSFs, angiogenin, CXC chemokines with ELR motif, insulin-like growth factor- (IGF-) 1, erythropoietin, and interleukin- (IL-) 8. Another was direct contact with vessel endothelial through integrins and VE-cadherin [26]. Coculture experiment showed that endostar significantly suppressed the proliferation of HUVEC cocultured with HepG2 and cultured in HCM alone. The suppression effective was more obvious in HUVEC cocultured with HepG2 than in HUVEC cultured in HCM alone, which suggested that endostar suppressed the proliferation of endothelial cell affected by multiple factors and induced by HCC cells.

Similar results were detected in tube formation assay. In one way, HCC cells improved the formation of tubes by secreting active factors such as TGF-*β*, TNF-*α*, and angiotropin [27]. In another way, HCC cells improved the formation of tubes via direct contact with PECAM-1 and VE-cadherin [28].

In Matrigel, the net tube structure was obviously decreased by the addition of endostar. When the concentration of endostar was between 50 ng/mL and 5000 ng/mL, the peak point of suppression was 50 ng/mL, which suggested endostar suppressed the tube structure formation of endothelial cell affected by multiple factors and induced by HCC cells.

Multiple cytokines such as VEGF-family, TNF*α*, EGF, angiotropin, and HGF were closely related to* in vivo* angiogenesis [29]. Our research results also showed that HCC cells could induce more blood vessels, while endostar significantly suppressed the induced angiogenesis. Detection of hemoglobin quantity in plugs showed that during the concentrations from 50 ng/mL to 5000 ng/mL, endostar could significantly suppress HCC induced angiogenesis dose dependently.

In summary, our study using a series modified classical* in vitro* experiments such as Boyden chambers migration array, wound-healing assay, cell adhesion assay, CFSE-labeled flow cytometric analysis, and tube formation assay analyzed the suppression effect of endostar on HCC-induced endothelial cell migration, adhesion, proliferation, and tube formation, which suggest that endostar could suppress the angiogenesis of HCC* in vitro*. We also analyzed the suppression effect of HCC-induced angiogenesis* in vivo* by modified Matrigel plug assay suppress, which showed that endostar could suppress the angiogenesis of HCC* in vivo*.

The molecular mechanism of endostar suppressing HCC-induced angiogenesis still needs further elucidation. However, our research confirmed that endostar had suppression effect towards multiple factor effect and HCC-induced angiogenesis. The results provide theoretical and experimental proofs for the role of endostar in antiangiogenesis therapy.
